# Process Optimization and Microstructure in High-Speed Coaxial Dual-Laser Welding of SUS301 Thin Sheets Using an SSA-BP Model

**DOI:** 10.3390/ma19122451

**Published:** 2026-06-08

**Authors:** Dexi Wang, Nan Li, Xiaohong Yan, Chunli Li, Hongyang Wang, Liming Liu

**Affiliations:** 1 School of Materials Science and Engineering, Dalian University of Technology, Dalian 116024, China; dxwang22@163.com (D.W.); linan_0819@mail.dlut.edu.cn (N.L.); lichunli2468@mail.dlut.edu.cn (C.L.); liulm@dlut.edu.cn (L.L.); 2 Dalian Area Representative Office, Dalian 116024, China; wannerman@126.com

**Keywords:** SUS301 stainless steel, coaxial dual-laser welding, SSA-BP

## Abstract

**Highlights:**

- SSA-BP improved BP prediction accuracy and showed stable performance in five-fold cross-validation.
- A favorable empirical aspect ratio interval of 0.82–0.84 was associated with stable weld formation and relatively high tensile strength.
- Fractography and EBSD phase/GND/KAM analyses revealed coordinated fracture and microstructural features in the representative high-strength joint.

**Abstract:**

To predict weld geometry and clarify structure–property relationships in high-speed coaxial dual-laser butt welding of 1 mm-thick SUS301 stainless steel sheets, an SSA-BP neural network model was established to describe the nonlinear correlation between welding parameters and weld morphology. The model related continuous laser power, welding speed, pulse frequency, and pulse width to weld width and penetration depth. To improve the transparency of model validation, conventional BP and SSA-BP models were compared using the same independent test set, and five-fold cross-validation was performed using the original experimental samples. On the independent test set, the SSA-BP model achieved an overall correlation coefficient of R = 0.960, with RMSE values of 0.0561 mm and 0.0439 mm for weld width and penetration depth, respectively. Compared with the conventional BP model, SSA-BP reduced the overall RMSE, MAE, and MAPE by 25.9%, 36.4%, and 29.6%, respectively. The five-fold cross-validation further indicated stable prediction performance under different data partitions. Based on the predicted and experimentally measured weld geometry, candidate parameter sets were screened according to the weld aspect ratio (Φ = h/w). Within the present experimental window, joints with Φ = 0.82–0.84 showed more stable weld formation and relatively higher ultimate tensile strength (1211.4–1264.8 MPa) than two representative joints outside this interval (796.0 MPa at Φ = 0.63 and 1061.1 MPa at Φ = 0.88). Therefore, this interval should be regarded as a favorable empirical range under the present welding conditions rather than a universal optimum. Fractographic observations of a representative high-strength joint showed abundant dimples and tear ridges, indicating ductile fracture characteristics. EBSD analysis further revealed a graded microstructure from the weld center to the base metal. The weld center and fusion line-adjacent regions exhibited relatively high fractions of high-angle grain boundaries (66.2–70.6%), while phase distribution, GND density, and KAM maps indicated a gradual phase transition and localized but non-continuous strain concentration features across the joint. These results indicate that the present approach provides an effective route for weld geometry prediction and for linking morphology screening with tensile response and microstructural heterogeneity in SUS301 thin sheet welding.

## 1. Introduction

With the escalating demand for lightweight, high-strength, and high-formability metallic components in global engineering sectors such as rail transit and automotive industries, SUS301 metastable austenitic stainless steel has become a preferred material for thin-walled load-bearing structures due to its exceptional combination of strength, ductility, and corrosion resistance [1,2]. As a typical ultrahigh-strength steel, SUS301 achieves superior specific strength through strain-induced martensitic transformation, demonstrating immense potential in aerospace applications pursuing extreme weight reduction [1]. Owing to its high specific strength and good formability, SUS301 stainless steel has been widely used in lightweight thin-walled structural components in transportation and aerospace-related engineering fields. SUS301 is a metastable austenitic stainless steel whose mechanical response is strongly associated with deformation-induced martensitic transformation. During welding, the rapid thermal cycle, local melting and solidification, and subsequent cooling may modify the original deformation-induced microstructure and affect the balance between austenite and martensitic transformation-related features. Therefore, for SUS301 thin sheets, weld quality should not be evaluated only by macroscopic bead geometry, but also by the microstructural heterogeneity generated by the welding thermal cycle. For thin sheets with a thickness of 1 mm or less, the quality of welded joints directly dictates the dimensional stability, service safety, and structural integrity of spacecraft and aviation components [3]. However, owing to the limited thickness and high sensitivity to heat input, thin sheet laser welding is highly susceptible to burn-through and burn-through hole defects, which seriously compromise weld formation quality and joint reliability [4].

To improve the limited molten pool stability and process controllability associated with conventional single-beam laser welding, dual-laser-assisted welding has attracted increasing attention. Möbus and Woizeschke [5] developed a laser welding setup for the coaxial combination of two laser beams and demonstrated that independently adjustable power distribution and focal plane positioning can effectively modify the axial intensity distribution, thereby significantly affecting weld penetration and process stability. More recently, Li et al. [6] proposed a coaxial continuous pulsed dual-beam laser process for Ti alloy/steel joining and showed that the dual-beam configuration produced better joint appearance and mechanical performance than single-beam laser welding, while also revealing the distinct roles of the continuous and pulsed lasers in interfacial bonding and fracture behavior. In the field of stainless steel joining, dual-beam laser welding has likewise been shown to reduce porosity and improve weld quality by enlarging the molten pool, enhancing fluid flow, and stabilizing keyhole behavior. These studies indicate that dual-beam and especially coaxial dual-beam laser welding provide an effective route for heat-input regulation and weld-quality improvement [7]. Nevertheless, the process still involves multiple strongly coupled variables, such as laser power, welding speed, pulse frequency, and pulse width, making it difficult for conventional trial-and-error or single-factor approaches to efficiently identify a reliable parameter window.

In parallel with the development of advanced welding processes, machine learning-based methods have gradually been introduced into welding research for process modeling, weld quality prediction, and parameter optimization [8]. The complexity of laser welding, characterized by highly nonlinear correlations between input parameters, weld-pool behavior, and final weld morphology, requires advanced computational prediction tools. Artificial neural networks are particularly suitable for this purpose because they can approximate nonlinear input–output relationships without requiring explicit analytical expressions. Previous studies in welding and mechanical engineering have shown that ANN-based models can provide reliable predictions of material behavior and process responses under variable operating conditions. Sathiya, Panneerselvam, and Abdul Jaleel [9,10] employed artificial neural networks combined with a genetic algorithm to optimize laser welding parameters for super austenitic stainless steel by simultaneously considering penetration depth, bead width, and tensile strength, demonstrating the capability of neural network-based methods to capture nonlinear relationships between welding inputs and multiple performance indicators. Subsequent studies further extended neural-network and deep learning methods to weld geometry prediction, penetration depth estimation, and multi-information-based weld quality assessment [11,12]. Recent reviews have also pointed out that machine learning has become an important tool in welding research, particularly for multiparameter process optimization and quality prediction [13,14]. However, for highly coupled processes with relatively limited datasets, conventional BP neural networks remain sensitive to the initial weights and thresholds and are prone to local optima, which may reduce convergence efficiency, robustness, and predictive accuracy [15]. Although GA and PSO have been widely used to optimize neural network models in welding applications, SSA was selected in this study because it provides a simple population-based search mechanism with discoverer–follower–vigilante updating. This mechanism is suitable for optimizing the initial weights and thresholds of BP neural networks and can improve convergence stability under limited and highly coupled datasets. Therefore, the SSA-BP framework was adopted as a practical compromise between prediction accuracy, algorithmic simplicity, and computational cost. More importantly, many studies mainly focus on geometric prediction itself, whereas the relationship between model-based parameter screening, joint load-bearing capacity, and microstructural heterogeneity remains insufficiently clarified.

In addition, previous studies on laser welding of stainless steels have shown that changes in process parameters can substantially alter weld pool geometry, solidification behavior, and final microstructure [16]. Patterson, Lippold, and Panton emphasized that weld geometry directly affects solidification conditions and therefore plays a critical role in microstructure evolution in stainless steel laser welds. This means that the selection of welding parameters should not be based solely on weld width or penetration depth, but should also consider whether the resulting geometric characteristics are compatible with favorable mechanical response and meaningful structure-property correlation [17,18]. This issue is particularly important for high-speed coaxial dual-laser welding of SUS301 thin sheets, for which both the parameter coupling and the microstructural heterogeneity are pronounced [3,19].

Based on the above analysis, this study focuses on high-speed coaxial continuous pulsed dual-laser welding of 1 mm thick SUS301 stainless-steel sheets. With the pulsed laser power fixed at 640 W, the effects of CW laser power, welding speed, pulse frequency, and pulse width on weld width, penetration depth, aspect ratio, and joint performance were investigated. An SSA-BP model was established to predict weld width and penetration depth, and its accuracy and stability were evaluated by comparison with conventional BP and five-fold cross-validation. The favorable empirical parameter range was then screened using weld aspect ratio and tensile performance, while SEM fractography, EBSD phase distribution, GND density, and KAM analyses were used to explain the fracture behavior and microstructural features of a representative high-strength joint. This work integrates weld geometry prediction, parameter screening, mechanical validation, and microstructural analysis to support process optimization for high-speed coaxial dual-laser welding of SUS301 thin sheets.

## 2. Materials and Methods

### 2.1. Materials and Welding System

The base material used in this study was SUS301 stainless steel sheet with dimensions of 100 mm × 50 mm × 1 mm. Its chemical composition is listed in Table 1. Before welding, the butt joint edges and adjacent surfaces were mechanically ground using fine abrasive paper to remove surface oxides, burrs, and loose contaminants. The specimens were then wiped with absolute ethanol to remove oil and residual surface impurities. No chemical etching treatment was applied before welding. A zero-gap butt joint configuration was adopted. During welding, the specimens were fixed using a dedicated fixture to suppress thermal deformation and ensure stable alignment.

**Figure 1:**
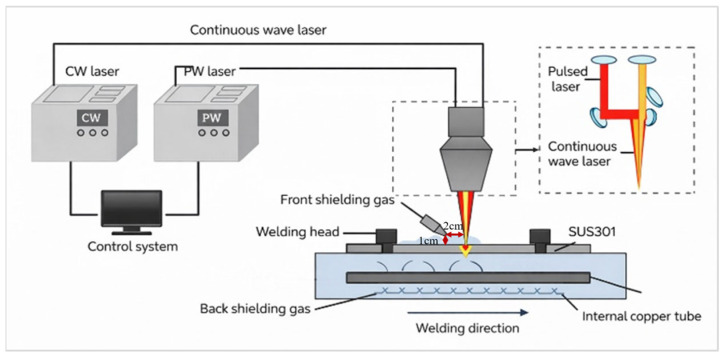
Schematic diagram of the coaxial dual-laser welding system.

The welding experiments were carried out using a coaxial dual-laser welding system composed of a continuous-wave (CW) laser and a pulsed laser, as shown in Figure 1. The two laser beams were coaxially combined and delivered to the same weld zone. The CW laser was primarily used to provide the main heat input required for weld penetration, whereas the pulsed laser was used to regulate the thermal behavior and dynamic stability of the molten pool. The maximum output power of the CW laser was 2000 W, with a wavelength of 980 nm and a minimum spot diameter of 0.8 mm. The pulsed laser had a maximum output power of 800 W and a wavelength of 1064 nm. In the present study, the pulsed laser power was fixed at 640 W, corresponding to 80% of its maximum output power. This value was selected based on preliminary welding trials. Lower pulsed laser power provided insufficient regulation of the molten pool, whereas excessively high pulsed laser power tended to increase bead instability. To reduce the dimensionality of the experimental design and focus on the coupled effects of CW laser power, welding speed, pulse frequency, and pulse width, the pulsed laser power was treated as a controlled parameter rather than an independent variable. During welding, the laser head was aligned with the centerline of the butt joint, and the welding path and speed were controlled by the welding platform and control program. The continuous laser power, welding speed, pulse frequency, and pulse width were set according to the experimental design.

### 2.2. Experimental Design and Characterization

To establish a dataset for welding parameter optimization, preliminary welding experiments were first conducted to investigate the effects of key process variables on weld geometric formation and joint performance. In this study, continuous laser power, welding speed, pulse frequency, and pulse width were selected as the main input variables, while weld width and penetration depth were used as the primary geometric response indicators. The pulsed laser power was kept constant at 640 W throughout the experiments.

Based on the preliminary experimental results, the investigated ranges of continuous laser power, welding speed, pulse width, and pulse frequency were limited to 800–1500 W, 1100–3200 mm/min, 4.0–6.4 ms, and 20–55 Hz, respectively. The pulsed laser power was excluded from the variable set because it was fixed at 640 W in all experiments. The specific parameter ranges are listed in Table 2. In the subsequent SSA-BP model, continuous laser power, welding speed, pulse frequency, and pulse width were taken as input variables, whereas the pulsed laser power was fixed at 640 W and was therefore not treated as an independent variable, whereas weld width and penetration depth were used as output variables. To reduce the influence of dimensional inconsistency on model training, the sample data were normalized before training.

**Figure 2:**
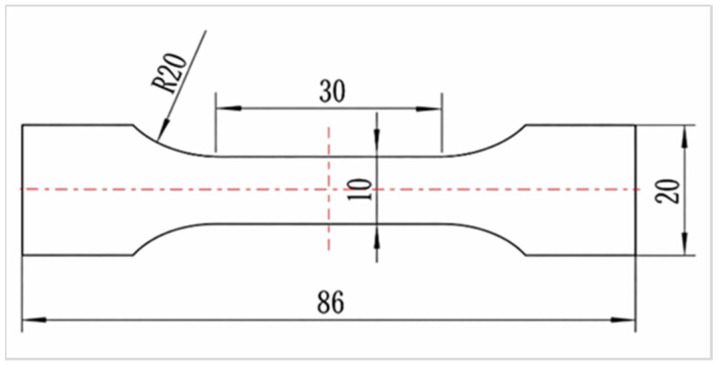
Geometry of the standard tensile specimen (Unit: mm).

After welding, the joints were subjected to mechanical testing as well as macroscopic and microscopic characterization. No in situ temperature measurement was performed during welding. Therefore, the present study does not aim to validate a thermal model directly. Instead, the thermal effect of the welding process was interpreted indirectly through weld morphology, tensile behavior, and EBSD-observed microstructural gradients. Future work will incorporate infrared thermography or thermocouple-assisted measurements to quantitatively correlate transient thermal histories with microstructural evolution. The dimensions of the tensile specimen are shown in Figure 2. Tensile tests were performed using a CSS-2205 universal testing machine at a loading speed of 2 mm/min, and the maximum fracture load and fracture location of the joint were recorded. To observe the weld cross-sectional morphology, the welded samples were sectioned perpendicular to the welding direction. After standard grinding, polishing, and etching, the weld geometry, microstructure, and fracture features were examined using optical microscopy and scanning electron microscopy. Typical regions of one representative joint selected from the favorable aspect ratio interval were further characterized by electron backscatter diffraction (EBSD) to analyze the microstructural differences among the weld center, fusion line, heat-affected zone, and base metal.

The original experimentally measured dataset consisted of 80 samples. For model development, only the training subset was augmented by noise injection, whereas all validation and test samples remained experimentally measured data. The augmented samples were not regarded as additional independent experiments, but were used only to improve the numerical stability of neural network training. In addition, five-fold cross-validation was performed on the original 80 experimental samples, and in each fold, noise injection was applied only to the training subset, respectively. Small random perturbations were introduced only into the training samples, and the perturbation amplitude for each input variable was controlled within a small proportion of its experimental range so as to preserve physical plausibility and avoid unrealistic parameter combinations. The augmented training set was then used for BP and SSA-BP model training.

## 3. Weld Geometry Prediction and Parameter Optimization

### 3.1. BP Neural Network

**Figure 3:**
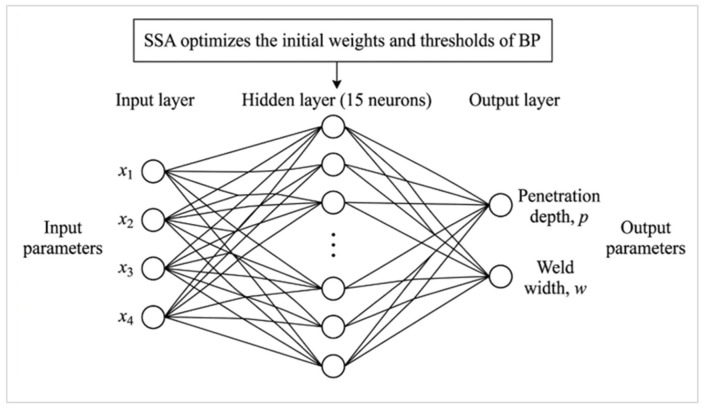
Schematic illustration of the neural network architecture.

During high-speed coaxial dual-laser welding, continuous laser power, welding speed, pulse frequency, and pulse width jointly determine the effective heat input and its temporal-spatial distribution under the condition of a fixed pulsed laser power, thereby exerting a significant coupled effect on weld width and penetration depth. Because a clear nonlinear relationship exists between welding process parameters and weld geometric features, a BP neural network was employed in this study to establish the mapping model between them [11]. The model takes continuous laser power *p*, welding speed *v*, pulse frequency *f*, and pulse width *τ* as input variables, and weld width *W* and penetration depth *h* as output variables. The network structure is shown in Figure 3.

To reduce the influence of dimensional inconsistency among variables on model training, the sample data were normalized using min–max normalization:

(1) $$x_{i}^{\prime }=\frac{x_{i}-x_{\min}}{x_{\max}-x_{\min}}$$

where $x_{i}$ is the original data, ${x^{\prime }}_{i}$ is the normalized data, and $x_{\min}$ and $x_{\max}$ are the minimum and maximum values of the variable in the sample set, respectively. Min–max normalization was used because all input variables were bounded within the experimentally designed process window. Nevertheless, this method is sensitive to outliers because the minimum and maximum values determine the scaling range. To reduce this influence, the dataset was checked before model training to exclude physically unrealistic parameter combinations and severely defective welds. Therefore, the established model should be regarded as valid mainly within the investigated process window, and extrapolation beyond this range should be treated with caution.

The BP neural network consists of an input layer, a hidden layer, and an output layer. Let *W_ij_* denote the connection weight from the input layer to the hidden layer, *a_j_* the threshold of the hidden layer, *V_jk_* the connection weight from the hidden layer to the output layer, and *b_k_* the threshold of the output layer. Then, the output of the *j*-th neuron in the hidden layer is:

(2) $$H_{j}=f(\sum_{i=1}^{m}w_{ij}x_{i}-a_{j})$$

and the output of the *k*-th neuron in the output layer is:

(3) $$Y_{k}=g(\sum_{j=1}^{l}v_{jk}H_{j}-b_{k})$$

where m is the number of input-layer nodes, l is the number of hidden-layer nodes, and f(⋅) and g(⋅) are the activation functions of the hidden and output layers, respectively. In this study, f(⋅) was the hyperbolic tangent sigmoid function (tansig), and g(⋅) was the linear transfer function (purelin), which is suitable for continuous regression outputs such as weld width and penetration depth.

The network was trained using the mean squared error (MSE) function as the objective function:

(4) $$E=\frac{1}{N}\sum_{n=1}^{N}\sum_{k=1}^{q}{\left(T_{nk}-Y_{nk}\right)}^{2}$$

where N is the number of samples, q is the number of output-layer nodes, $T_{nk}$ is the target value of the *k*-th output for the *n*-th sample, and $Y_{nk}$ is the corresponding predicted value.

In this study, the BP neural network model was established on the MATLAB 2024b platform. After parameter comparison, the network structure was determined to be 4-15-2, where the numbers of nodes in the input, hidden, and output layers were 4, 15, and 2, respectively. The learning rate was set to 0.01, and the target training error was set to 10^−3^. On this basis, the BP neural network was used to achieve nonlinear fitting between welding process parameters and weld width/penetration depth.

### 3.2. SSA-Optimized BP Neural Network

In a conventional BP neural network, the initial weights and thresholds are usually assigned randomly. As a result, the training outcome is highly sensitive to these initial values, and problems such as slow convergence and convergence to local optima may arise. To improve training stability and prediction accuracy, the sparrow search algorithm (SSA) was employed in this study to optimize the initial weights and thresholds of the BP neural network, thereby constructing an SSA-BP prediction model. The SSA-BP model mainly consists of three stages: determination of the BP network structure, global optimization by SSA, and BP network training [20].

The total dimensionality *D* of the SSA optimization vector includes all connection weights and bias terms of the BP network. For a fully connected network with *m* input neurons, *l* hidden neurons, and *q* output neurons, *D* is expressed as:

(5) D=m×l+l×q+l+q

where *m* × *l* represents the weights between the input and hidden layers, *l* represents the hidden-layer bias terms, *l* × *q* represents the weights between the hidden and output layers, and *q* represents the output-layer bias terms. For the present 4-15-2 network, *D* = 4 × 15 + 15 + 15 × 2+2 = 107.

In the SSA-BP model, the initial weights and thresholds of the BP network are encoded as the positions of sparrow individuals, and the mean squared error of the BP network is taken as the fitness function:

(6) Fitness=E

That is, the combination of weights and thresholds that minimizes the error function is searched to obtain more favorable initial parameters for the BP network.

In SSA, discoverers are responsible for searching for food and guiding the position update of the population [20]. Their position update formula can be expressed as:

(7) $$X_{i,j}^{t+1}={\{}_{X_{i,j}^{t}+Q\cdot L,\;\;\;\;\;\;\;\;\;\;\;\;\;\;\;\;\;\;\;\;\;\;R_{2}\geq ST}^{X_{i,j}^{t}\exp(-\frac{i}{\alpha T_{\max}}),\;\;\;\;\;R_{2}<ST}$$

where $X_{i,j}^{t}$ denotes the position of the *i*-th sparrow in the *j*-th dimension at generation *t*, *T_max_* is the maximum number of iterations; *a* is a constant; *R*_2_ is the warning value; *ST* is the safety threshold; *Q* is a random number obeying a normal distribution; and *L* is a matrix with all elements equal to 1.

Followers update their positions according to the positions of the discoverers, and the update formula is:

(8) $$X_{i,j}^{t+1}={\{}_{X_{p}^{t+1}+\left|X_{i,j}^{t}-\left.X_{p}^{t+1}\right|\cdot A^{+}\cdot L,i\leq \frac{n}{2}\right.}^{Q\cdot \exp(\frac{X_{worst}^{t}-X_{i,j}^{t}}{n}),i>\frac{n}{2}}$$

where $X_{worst}^{t}$ is the current worst position, $X_{p}^{t+1}$ is the current best discoverer position, and *A^+^* is the generalized inverse of matrix *A*.

When some individuals in the population become aware of danger, the vigilantes adjust their positions according to:

(9) $$X_{i,j}^{t+1}={\{}_{X_{i,j}^{t}+K\cdot \frac{\left|X_{i,j}^{t}-\left.X_{worst}^{t}\right|\right.}{(f_{i}-f_{w})+\varepsilon },f_{i}=f_{g}}^{X_{best}^{t}+\beta \left|X_{i,j}^{t}-\left.X_{best}^{t}\right|,f_{i}>f_{g}\right.}$$

where $X_{best}^{t}$ and $X_{worst}^{t}$ are the current global best and worst positions, respectively; *β* is the step-size control parameter; *K* is a random number within [−1,1]; *ε* is a random constant; and $f_{i}$, $f_{g}$, and $f_{w}$ are the fitness values of the current individual, the global best, and the global worst, respectively.

**Figure 4:**
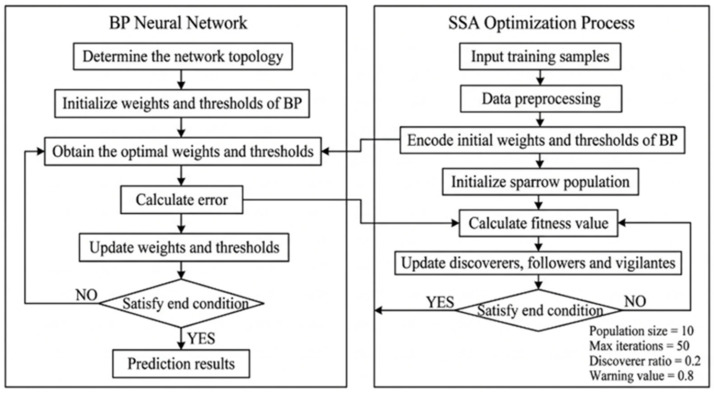
Flowchart of the SSA-BP optimization procedure.

Flowchart of the SSA-BP optimization procedure is shown in Figure 4. The SSA parameters used in this study were as follows: population size of 10, maximum number of iterations of 50, discoverer proportion of 0.2, and warning value of 0.8. The optimization flow of the SSA-BP model is shown in Figure 4. First, the topology of the BP network was determined according to the input and output variables, and the sample data were normalized. Second, the initial weights and thresholds of the BP network were encoded as sparrow positions, and the sparrow population was initialized. Then, with the mean squared error as the fitness function, the population was iteratively optimized according to the SSA position update mechanism. After the maximum number of iterations was reached or the convergence criterion was satisfied, the weights and thresholds corresponding to the optimal individual were output. Finally, the optimized results were assigned to the BP neural network for training, thereby enabling the prediction of weld width and penetration depth.

### 3.3. Relationship Between Aspect Ratio and Joint Ultimate Tensile Strength

To comprehensively characterize the cross-sectional morphology of the weld, the aspect ratio Φ was defined as the ratio of penetration depth *h* to weld width *w*:

(10) $$\Phi =\frac{h}{w}$$

The aspect ratio can, to some extent, reflect the narrow-and-deep feature and geometric coordination of the weld cross-section, and is one of the important parameters for evaluating weld morphology [21]. For the high-speed coaxial dual-laser butt welding of 1 mm-thick SUS301 stainless steel sheets, weld penetration depth and width not only determine whether stable penetration can be achieved, but also affect heat input distribution and the final tensile performance. The experimental parameters and results for the 40 validation sets are shown in Table 3.

**Figure 5:**
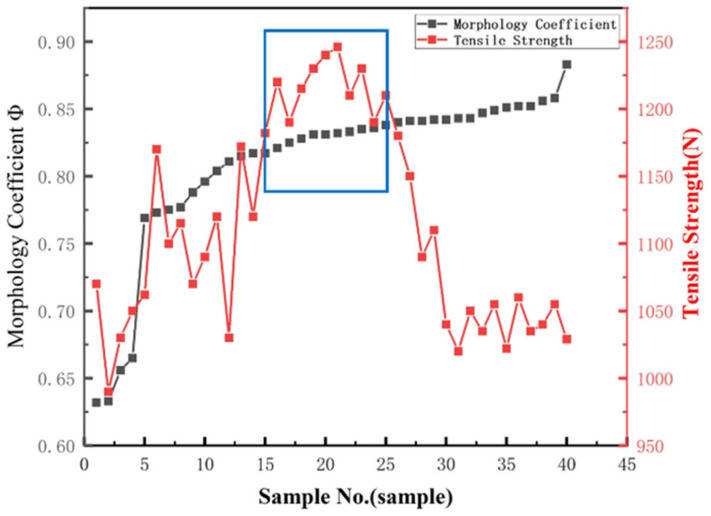
Relationship between ultimate tensile strength and weld aspect ratio.

It can be seen in Figure 5 that the ultimate tensile strength of the joint does not increase monotonically with changes in aspect ratio, but instead shows a trend of first increasing and then decreasing. This indicates that the highest joint UTS usually corresponds to an aspect ratio within a certain range, rather than the largest possible aspect ratio. When the aspect ratio is too small, the weld may suffer from insufficient penetration or poor morphological coordination. In contrast, when the aspect ratio is excessively large, the weld cross-section tends to become narrow and deep, causing local heat input concentration, which may be detrimental to weld formation stability [22].

**Figure 6:**
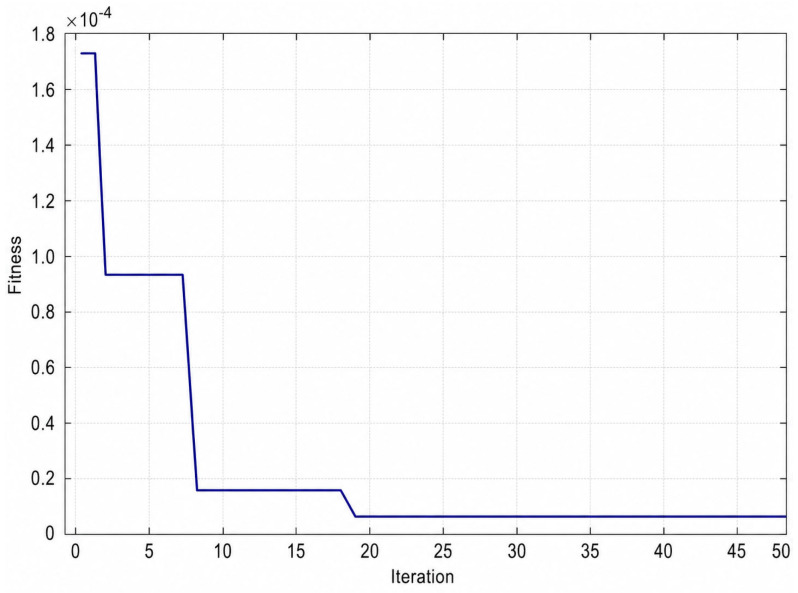
Iterative fitness curve of the sparrow search algorithm.

As shown in Figure 6, the SSA fitness curve shows that the fitness value decreased rapidly in the early stage of iteration and then gradually stabilized, indicating that the sparrow search algorithm could effectively search the initial weights and thresholds of the BP network within a limited number of iterations and enable the model to enter a stable convergence state relatively quickly. Under the conditions of a population size of 10 and a maximum of 50 iterations, SSA effectively optimized the initial network parameters.

### 3.4. Analysis of Model Training Results

**Figure 7:**
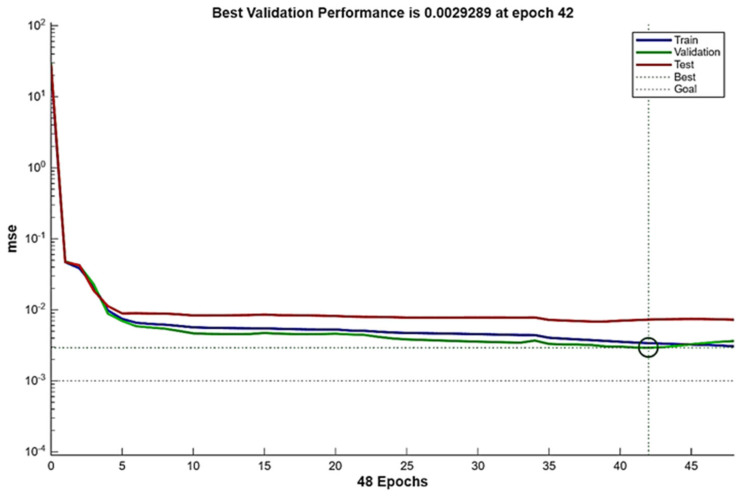
Convergence behavior of the SSA-BP neural network.

Figure 7 shows the best validation performance occurred at the 43rd epoch, corresponding to a validation error of 0.0029289. These results indicate that the predicted values of the SSA-BP model are well correlated with the measured values, and that the model can effectively describe the nonlinear mapping relationship between welding process parameters and weld width/penetration depth.

To quantitatively evaluate the prediction performance of the SSA-BP model, the correlation coefficient R, root mean square error RMSE, mean absolute error MAE, and mean absolute percentage error MAPE were calculated as follows:

(11) $$R=\frac{\sum_{i=1}^{N}\left(y_{i}-\bar{y})({\hat{y}}_{i}-\bar{\hat{y}}\right)}{\sqrt{\sum_{i=1}^{N}{\left(y_{i}-\bar{y}\right)}^{2}\sum_{i=1}^{N}{\left({\hat{y}}_{i}-\bar{\hat{y}}\right)}^{2}}}$$

where $y_{i}$ is the measured value, ${\hat{y}}_{i}$ is the predicted value, and $\bar{y}$ and $\bar{\hat{y}}$ are the mean values of the measured and predicted values, respectively. The closer *R* is to 1, the better the model fitting performance.

Meanwhile, the prediction error of the model can be expressed by the root mean square error:

(12) $$RMSE=\sqrt{\frac{1}{N}\sum_{i=1}^{N}{\left(y_{i}-{\hat{y}}_{i}\right)}^{2}}$$

(13) $$MAE=\frac{1}{N}\sum_{i=1}^{N}\left|y_{i}-\left.{\hat{y}}_{i}\right|\right.$$

The absolute error and relative error of a single sample can be expressed as:

(14) $$e_{i}=y_{i}-{\hat{y}}_{i}$$

(15) $$\delta_{i}=\frac{\left|y_{i}-\left.{\hat{y}}_{i}\right|\right.}{y_{i}}\times 100\%$$

**Figure 8:**
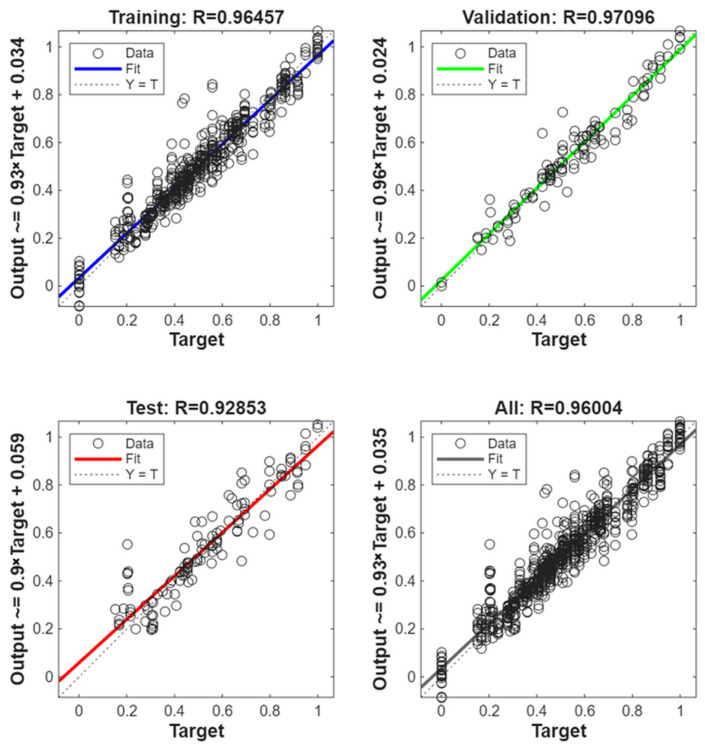
Regression analysis of the SSA-BP model.

As shown in Figure 8, the model regression results showed that the correlation coefficients of the training, validation, and test sets were 0.96457, 0.97096, and 0.92853, respectively, with an overall correlation coefficient of 0.96004. Taken together, the fitness curve, regression results, and error distribution indicate that the established SSA-BP model has good convergence and fitting capability within the current process window, and can be used for the preliminary screening of candidate parameter combinations [12,23]. Combined with the relationship between aspect ratio and tensile load, the parameter search range can be further narrowed, thereby providing a basis for subsequent welding experimental validation.

**Figure 9:**
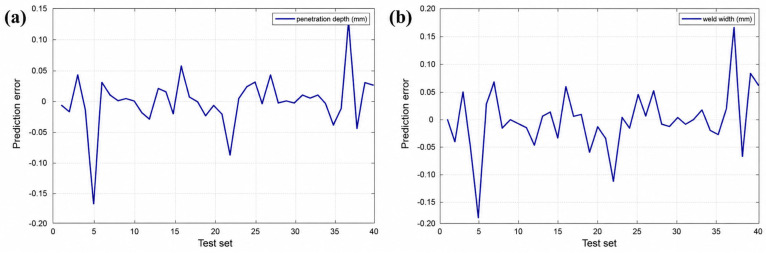
Prediction errors of the test set: (**a**) penetration depth error; (**b**) weld width error.

Figure 9 compares the prediction errors with the experimental results. These 40 sample groups were not included in the training set, ensuring the independence of the test data and enabling validation of the model’s generalization capability. The test samples covered different combinations of welding process parsameters in order to evaluate the prediction accuracy of the model under diverse welding conditions. Except for a few individual samples, the prediction errors of most test samples were small, indicating that the model has good predictive capability for both weld width and penetration depth.

To quantitatively evaluate the prediction performance and clarify the incremental contribution of SSA optimization, the conventional BP model and the SSA-BP model were compared using the same input variables, output variables, normalization procedure, and independent test set. The evaluation metrics included R, RMSE, MAE, and MAPE.

As shown in Table 4, compared with the conventional BP model, the SSA-BP model reduced the overall RMSE from 0.0680 mm to 0.0504 mm, the overall MAE from 0.0508 mm to 0.0323 mm, and the overall MAPE from 4.39% to 3.09%. The corresponding reductions were 25.9%, 36.4%, and 29.6%, respectively. The improvement was more pronounced for weld width prediction, with reductions of 31.1% in RMSE, 39.6% in MAE, and 33.6% in MAPE. For penetration depth prediction, although the correlation coefficient of SSA-BP was comparable to that of BP, the RMSE, MAE, and MAPE were all reduced. These results indicate that SSA optimization improved the initialization of BP weights and thresholds and reduced the prediction error of the neural network within the present process window.

To further evaluate the robustness of the SSA-BP model and reduce the dependence on a single train–validation–test split, five-fold cross-validation was performed using the original 80 experimentally measured samples. In each fold, four-fifths of the original samples were used for training and the remaining one-fifth was used for validation. Noise injection was applied only to the training subset, whereas the validation subset was kept as original experimental data to avoid information leakage. The weld aspect ratio Φ was not used as an input variable. The cross-validation results are summarized in Table 5.

As shown in Table 6, the five-fold cross-validation results indicate that the SSA-BP model maintained stable prediction performance under different data partitions. The mean RMSE values for penetration depth and weld width were 0.0305 mm and 0.0301 mm, respectively, while the corresponding MAE values were 0.0231 mm and 0.0228 mm. The mean overall MAPE was 2.08%, with a standard deviation of 0.48%. These results suggest that the prediction performance of the SSA-BP model was not dominated by a single favorable train–validation–test split. Nevertheless, because the number of original experimental samples was limited, the model should still be interpreted as a process window-specific prediction tool rather than a universally generalized model, the model should still be interpreted as a process window-specific prediction tool rather than a universally generalized model.

## 4. Discussion

### 4.1. Macroscopic Morphology and Tensile Properties of Welds

As shown in Figure 5, the joint tensile performance was relatively favorable when the weld aspect ratio was in the range of 0.82–0.84. Parameter screening should not simply target the extreme value of penetration depth or weld width alone; instead, under the premise of ensuring continuous weld formation and sufficient penetration, the weld aspect ratio should be maintained within a favorable range so as to obtain higher joint UTS and more stable tensile behavior [9,21]. Accordingly, samples 1–3 were selected from this interval for subsequent validation, and their aspect ratios were 0.82, 0.83, and 0.84, respectively. For comparison, samples 4 and 5 were selected from outside this interval, with aspect ratios of 0.63 and 0.88, In particular, the joint with Φ = 0.63 exhibited a sharp decrease in UTS to 796.0 MPa. This decrease can be mainly attributed to the poor coordination between penetration depth and weld width. A relatively low aspect ratio indicates a wide and shallow weld profile, which may reduce the effective load-bearing section and promote stress concentration near the fusion boundary during tensile loading. From a microstructural perspective, such a weld geometry usually corresponds to a different thermal cycle from that of the joints within the favorable aspect ratio range. The wider weld profile suggests a larger lateral heat-affected region and less concentrated penetration behavior, which may promote local softening and heterogeneous deformation near the fusion boundary or heat-affected zone. Although EBSD was not performed on this particular sample, its inferior macroscopic morphology and tensile response suggest that the corresponding solidification condition and thermal history were less favorable than those of the joints within Φ = 0.82–0.84. The specific experimental parameters are shown in Table 7. These representative samples were comparatively analyzed in terms of macroscopic weld morphology and engineering stress–strain behavior in order to verify the effectiveness of the aspect ratio-based screening criterion. In addition, EBSD analysis was performed on one representative joint selected from within the favorable aspect ratio interval so as to further elucidate the relationship among weld geometry, local microstructural evolution, and joint mechanical response.

**Figure 10:**
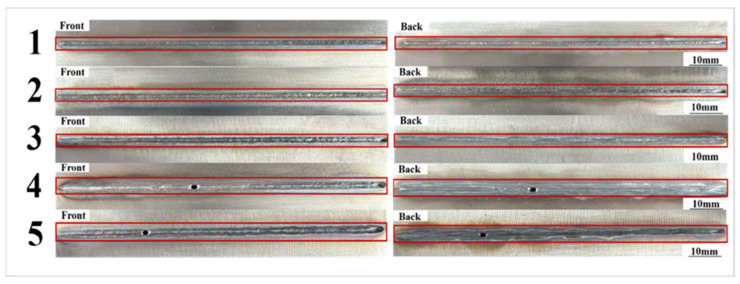
Surface appearance and cross-sectional morphology of welded joints produced using representative parameter sets.

Figure 10 shows the macroscopic surface and cross-sectional morphologies of samples 1–3, which were selected from within the favorable aspect ratio interval identified from Figure 5. The aspect ratios of these three joints were 0.82, 0.83, and 0.84, respectively. It can be seen that the weld surfaces were continuously formed and the weld beads were relatively uniform. In addition, the cross-sections exhibited complete profiles and clear fusion boundaries, without obvious macroscopic defects such as burn-through, lack of fusion, or severe collapse. These results indicate that when the aspect ratio is maintained within the range of 0.82–0.84, the weld formation is relatively stable under the present high-speed coaxial dual-laser welding conditions.

Porosity is a common defect in stainless-steel laser welding, especially when keyhole instability, vapor entrapment, or insufficient shielding occurs. In the present study, no obvious macroscopic pores were observed in the optical cross-sections of the representative joints within the favorable aspect ratio range. However, because X-ray radiography or micro-CT characterization was not performed, the existence of fine internal pores cannot be completely excluded. This limitation should be considered when applying the present results to industrial quality control.

It also presents the corresponding macroscopic morphologies of samples 4 and 5, which were selected from outside the favorable aspect ratio interval. Their aspect ratios were 0.63 and 0.86, respectively. Compared with samples 1–3, these joints showed relatively poorer geometric coordination and less satisfactory formation quality, suggesting that deviation from the favorable aspect ratio interval is detrimental to weld formation stability.

**Figure 11:**
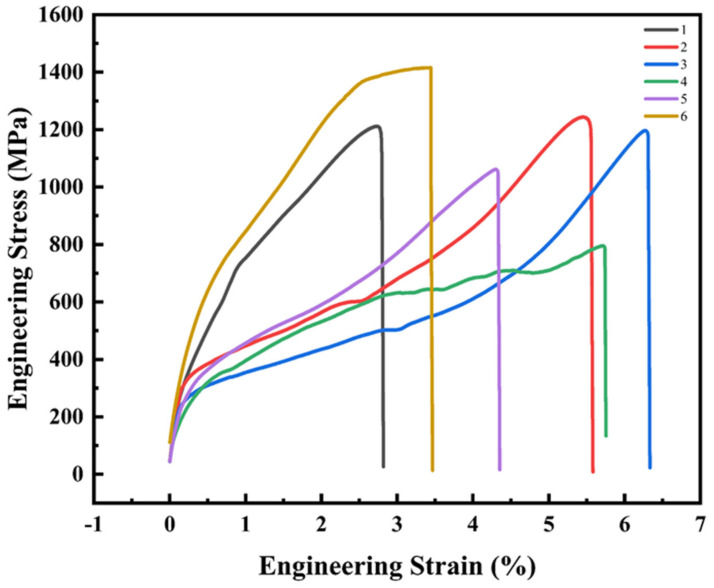
Engineering stress–strain curves.

The engineering stress–strain curves of samples 1–5 and the base metal are shown in Figure 11. Overall, samples 1–3 exhibited a more stable tensile response and better load-bearing behavior than samples 4 and 5 [24]. In addition, although the ultimate tensile strengths of all welded joints were lower than that of the base metal (1400.1 MPa), samples 1–3 still reached 1211.4–1264.8 MPa, with the highest value reaching more than 90% of the base-metal strength, whereas samples 4 and 5 showed much lower values (796.0 MPa and 1061.1 MPa, respectively). Combined with the morphology results, this comparison further indicates that the aspect ratio range of 0.82–0.84 corresponds to a more favorable balance between weld formation and mechanical performance.

The above comparison indicates that the joints selected within the favorable aspect ratio interval exhibited not only more coordinated weld morphology but also a more reliable tensile response. However, the mechanical behavior of a welded joint is governed not only by macroscopic weld geometry, but also by the local microstructural evolution induced by the welding thermal cycle. Therefore, further microstructural characterization was carried out on one representative joint selected from the favorable aspect ratio interval to clarify the metallurgical basis underlying the observed mechanical response.

**Figure 12:**
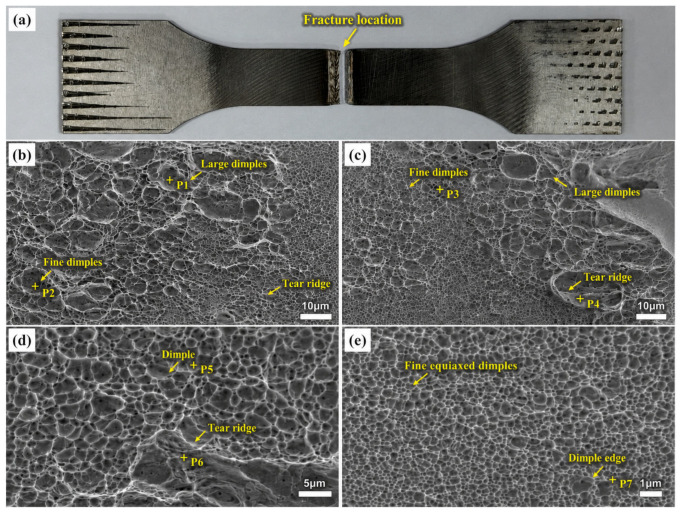
Fracture location and SEM fracture morphology of a representative high-strength joint: (**a**) fracture location; (**b**–**e**) SEM fracture surfaces.

The selected joint was located within the favorable empirical aspect ratio interval of Φ = 0.82–0.84 and exhibited relatively high tensile strength. As shown in Figure 12a, fracture occurred in the welded joint region, indicating that the tensile response was closely related to the local weld geometry and microstructural heterogeneity. The SEM fracture surfaces in Figure 12b–e show abundant dimples with different sizes, together with local tear ridges and dimple edges. The large dimples marked by P1 and P3 suggest microvoid growth around local heterogeneities, whereas the fine dimples marked by P2 and P7 indicate microvoid coalescence during plastic deformation. The tear ridges marked by P4 and P6 further suggest localized plastic deformation and microvoid linkage during tensile loading. These fractographic features indicate that the representative high-strength joint mainly exhibited ductile fracture characteristics rather than brittle cleavage fracture. Therefore, the fractographic observations provide supplementary evidence for the relatively stable tensile response of the selected joint.

### 4.2. Microstructure

**Figure 13:**
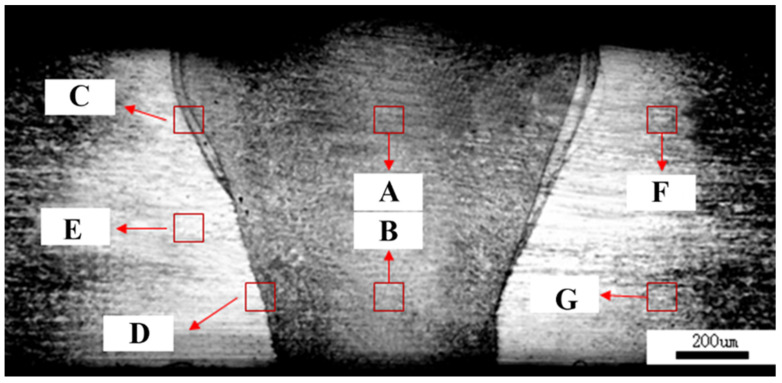
Metallographic cross-section of the weld and EBSD sampling locations. The analyzed area was divided into seven regions, including the upper and lower parts of the weld center (A and B), the upper and lower parts of the fusion line (C and D), the heat-affected zone (E), and the upper and lower parts of the base metal (F and G).

To further interpret the microstructural basis for the favorable tensile response observed in Section 4.1, EBSD characterization was performed on a representative joint selected from within the favorable aspect ratio interval. As shown in Figure 13, the analyzed area was divided into seven regions, including the upper and lower parts of the weld center (A and B), the upper and lower parts of the fusion line (C and D), the heat-affected zone (E), and the upper and lower parts of the base metal (F and G). This region selection captures both the transverse microstructural gradient from the weld center to the base metal and the possible difference in thermal history along the thickness direction.

To explain why the joint within the favorable empirical aspect ratio interval exhibited relatively high tensile strength and stable weld formation, EBSD analysis was carried out on a representative high-strength joint. Seven regions were selected across the joint, as shown in Figure 13: regions A and B correspond to the weld center, regions C and D are adjacent to the fusion line, region E is located in the heat-affected zone, and regions F and G correspond to the base metal. The purpose of this analysis was to determine whether the joint with favorable macroscopic morphology also showed a continuous and coordinated microstructural transition across the weld [25].

**Figure 14:**
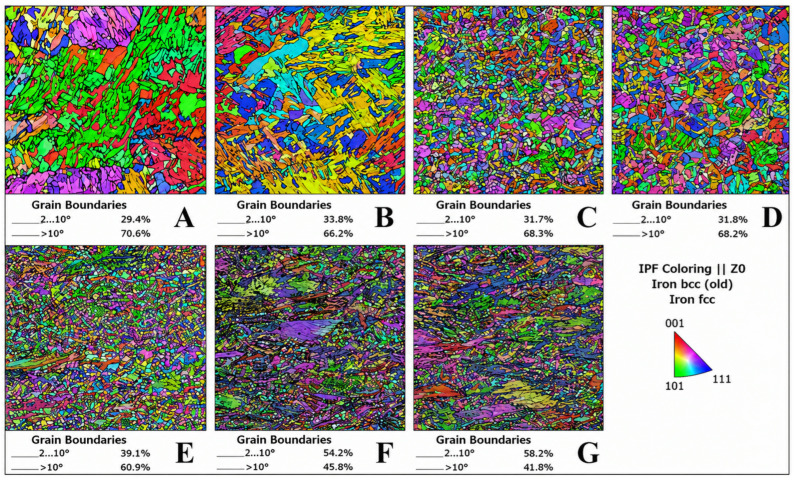
EBSD IPF maps and grain boundary distributions of the representative high-strength joint: (**A**,**B**) weld center; (**C**,**D**) fusion line-adjacent regions; (**E**) heat-affected zone; (**F**,**G**) base metal.

Figure 14 shows the IPF maps and grain boundary distributions. The weld center and fusion line-adjacent regions show relatively high fractions of high-angle grain boundaries. The HAGB fractions in regions A, B, C, and D are 70.6%, 66.2%, 68.3%, and 68.2%, respectively. These values are clearly higher than those in the base metal regions F and G, where the HAGB fractions decrease to 45.8% and 41.8%. This result indicates that the weld center and fusion line-adjacent regions underwent strong microstructural reconstruction during welding. More importantly, the HAGB fraction does not drop abruptly from the weld center to the fusion line-adjacent regions. Instead, A–D all maintain HAGB fractions above 66%, suggesting that the weld metal and fusion line regions formed a relatively continuous reconstructed microstructure. This is beneficial for avoiding a sharp weak boundary between the weld metal and the surrounding material.

Region E, corresponding to the HAZ, shows an intermediate HAGB fraction of 60.9%. This value is lower than that in the weld center but higher than that in the base metal. Therefore, the HAZ acts as a transition region rather than an abrupt discontinuity. From an engineering perspective, this is important because tensile failure is often promoted by sudden changes in microstructure and local mechanical response. In the present joint, the grain boundary data show a gradual transition from the reconstructed weld center to the deformation-retained base metal. This supports the observation that the joint within Φ = 0.82–0.84 maintained relatively high tensile strength.

**Figure 15:**
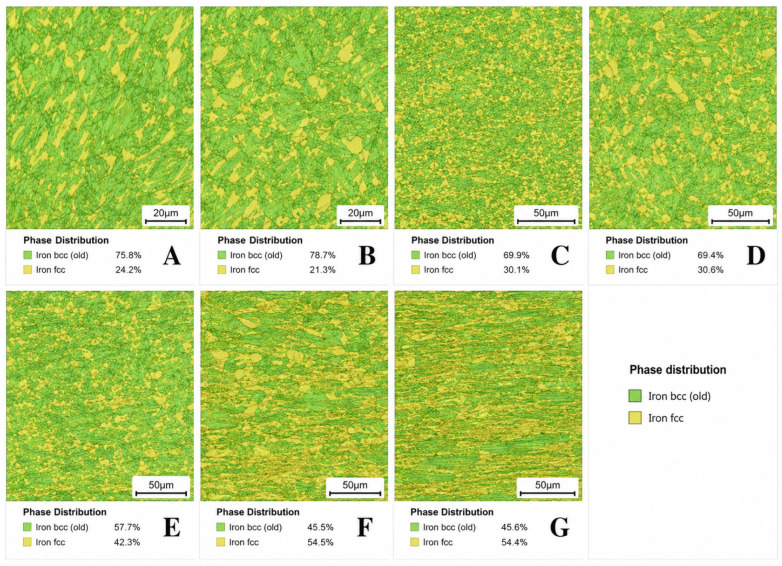
EBSD phase distribution maps of the representative high-strength joint: (**A**,**B**) weld center; (**C**,**D**) fusion line-adjacent regions; (**E**) heat-affected zone; (**F**,**G**) base metal.

Figure 15 presents the phase distribution maps. The phase fractions also show a clear gradient across the joint. In the weld center regions A and B, the bcc-indexed phase fractions are 75.8% and 78.7%, while the fcc fractions are 24.2% and 21.3%, respectively. In the fusion line-adjacent regions C and D, the bcc-indexed phase fractions decrease to 69.9% and 69.4%, and the fcc fractions increase to 30.1% and 30.6%. Moving further to the HAZ and base metal, the fcc fraction increases further: region E contains 42.3% fcc phase, while regions F and G contain 54.5% and 54.4% fcc phase, respectively.

This phase distribution result is important for explaining the joint performance. The weld center and fusion line regions retain a relatively high fraction of bcc-indexed phase, which is consistent with their relatively high local strength. At the same time, the fcc fraction increases gradually toward the base metal, which helps the surrounding material accommodate tensile deformation. Therefore, the phase distribution is not random; it shows a continuous transition from the weld center to the base metal. This supports the idea that the favorable aspect ratio did not only produce a suitable weld shape, but also corresponded to a more coordinated phase distribution across the joint.

**Figure 16:**
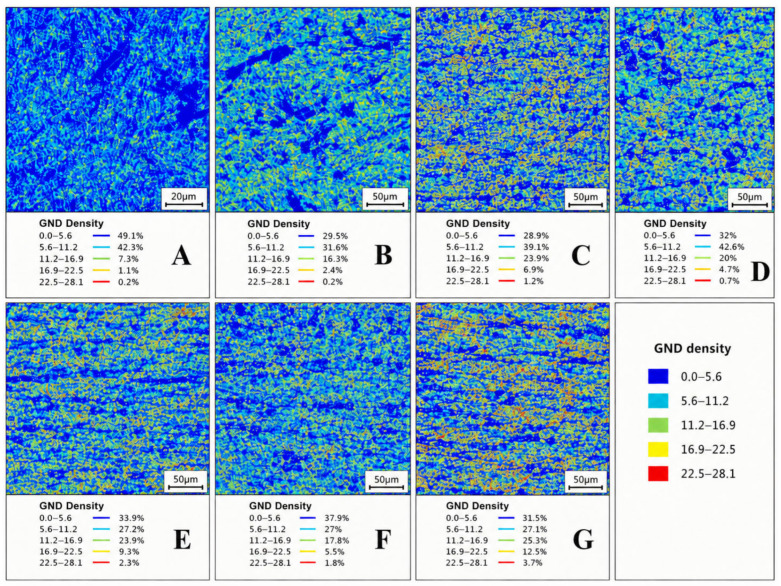
GND density maps of the representative high-strength joint: (**A**,**B**) weld center; (**C**,**D**) fusion line-adjacent regions; (**E**) heat-affected zone; (**F**,**G**) base metal.

Figure 16 shows the GND density maps. The GND distribution is also different among the selected regions. In regions A–D, the high-GND-density areas are mainly distributed locally along microstructural boundaries and band-like features, rather than forming a continuous high-density band across the entire weld. This is an important observation. If high-GND-density regions were continuously concentrated along the fusion line, they could easily become preferred paths for strain localization and crack propagation. In the present joint, however, the GND concentration is locally distributed, indicating that local deformation incompatibility was dispersed in different regions.

The GND data also show that the base metal and HAZ retain deformation-related features. Regions E–G contain more obvious band-like GND distributions, which is consistent with the higher low-angle grain boundary fractions observed in Figure 14. This means that the base metal still retains part of the rolling-induced deformation structure, while the weld center is more strongly reconstructed. The transition from weld metal to base metal is therefore not a single abrupt change, but a combination of reconstructed weld structure, transitional HAZ structure, and retained base-metal deformation structure. This microstructural continuity is consistent with the relatively stable tensile response of the selected high-strength joint.

**Figure 17:**
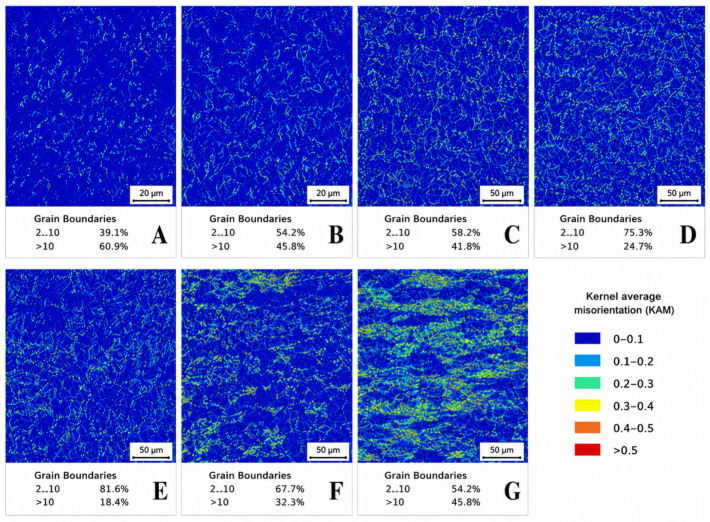
KAM maps of the representative high-strength joint: (**A**,**B**) weld center; (**C**,**D**) fusion line-adjacent regions; (**E**) heat-affected zone; (**F**,**G**) base metal.

Figure 17 shows the KAM maps. Most areas are dominated by low KAM values, while higher KAM values appear locally near boundaries and band-like features. This indicates that the joint does not contain widespread severe local misorientation after welding. In other words, the selected joint does not show large-area strain concentration in the EBSD maps. Instead, the local misorientation is distributed in limited areas. This result agrees with the GND maps and supports the relatively good tensile behavior of the joint. The absence of a continuous high-KAM band across the weld or fusion line region suggests that there was no obvious continuous weak zone in the analyzed high-strength joint.

Combining Figure 14, Figure 15, Figure 16 and Figure 17, the microstructural characteristics of the representative high-strength joint can be summarized as follows. First, the weld center and fusion line-adjacent regions show high HAGB fractions of 66.2–70.6%, indicating sufficient microstructural reconstruction in the weld region. Second, the HAZ has an intermediate HAGB fraction of 60.9%, forming a transition between the weld metal and base metal. Third, the phase distribution changes gradually from bcc-indexed-phase-rich weld regions to fcc-richer base metal regions, indicating that the phase distribution across the joint is continuous rather than abruptly separated. Fourth, the GND and KAM maps show localized but non-continuous strain concentration features, suggesting that local deformation can be distributed across multiple microstructural regions instead of concentrating along one weak path.

These EBSD results provide microstructural support for the high tensile strength of the selected joint within the favorable aspect ratio interval. The favorable macroscopic weld geometry reduced severe geometric stress concentration, while the EBSD results show that the corresponding microstructure also had a gradual transition in grain boundary character, phase distribution, GND density, and local misorientation. Therefore, the relatively high strength of this joint can be attributed to the combined effect of suitable weld morphology and coordinated microstructural transition.

It should be noted that the present EBSD analysis was performed on one representative high-strength joint within the favorable empirical aspect ratio interval. Therefore, these results provide direct microstructural evidence for the selected joint, but they should not be regarded as statistical proof that Φ = 0.82–0.84 is universally optimal. Additional EBSD comparison with low-strength joints outside this interval, such as Φ = 0.63 and Φ = 0.88, will be needed in future work to further confirm the relationship between aspect ratio, microstructure, and tensile performance.

## 5. Conclusions

This study established an SSA-BP model for high-speed coaxial dual-laser butt welding of 1 mm thick SUS301 stainless-steel sheets to predict weld width and penetration depth and to screen process parameters. Based on the model prediction, weld aspect ratio, tensile performance, and EBSD analysis were further combined to validate the weld formation, joint strength, and microstructural features within the selected parameter range. Overall, the results confirmed the initial expectation that an SSA-BP model can effectively predict weld geometry and assist parameter screening for high-speed coaxial dual-laser welding of SUS301 thin sheets. However, the results also showed that joint performance did not increase monotonically with penetration depth or aspect ratio. Instead, an appropriate aspect ratio window was required to balance weld formation and tensile response.

1. The established SSA-BP model effectively predicted weld width and penetration depth within the investigated process window in high-speed coaxial dual-laser welding of SUS301 sheets, with an overall correlation coefficient of 0.960.
2. Joint load-bearing capacity was strongly correlated with the weld aspect ratio. A favorable empirical range of Φ = 0.82–0.84 was associated with relatively stable weld formation and comparatively high tensile strength.
3. EBSD analysis of the representative high-strength joint revealed a graded microstructure from the weld center to the base metal. The weld center and fusion line-adjacent regions showed high HAGB fractions of 66.2–70.6%, while the phase distribution, GND, and KAM maps further indicated a gradual phase transition and localized but non-continuous strain concentration features. These microstructural characteristics provide supporting evidence for the relatively stable tensile response of the selected joint.
4. From an industrial perspective, the proposed SSA-BP-assisted screening method can reduce trial-and-error experiments and provide preliminary guidance for high-speed coaxial dual-laser welding of thin SUS301 stainless-steel sheets. However, the present model was developed using 1 mm thick SUS301 sheets within a specific process window. Therefore, when the sheet thickness, joint configuration, shielding condition, or laser power range changes, the model should be retrained or recalibrated using additional experimental data before industrial application.

## Figures and Tables

**Table 1:** Chemical composition of SUS301 stainless steel (wt.%).

Components	Fe	Cr	Ni	Mn	Cu	Co	Si	V	Mo	P
Content	74.11	16.98	5.07	1.74	0.43	0.35	0.34	0.10	0.84	0.04

**Table 2:** Range of welding experimental parameters.

Welding Parameters	Values
Continuous laser power (W)	800–1500
Welding speed (mm/min)	1100–3200
Pulse width (ms)	4.0–6.4
Pulse frequency (Hz)	20–55
Front shielding gas flow rate (L/min)	25
Back shielding gas flow rate (L/min)	40

**Table 3:** Sensitivity analysis of the hidden-layer size.

Hidden Neurons	Model Behavior
5	The hidden layer was too small, and the nonlinear mapping capability was insufficient.
8	The validation error decreased compared with the 5-neuron structure.
10	The model fitting capability continued to improve.
12	The validation error further decreased and approached a stable level.
15	The best validation performance was obtained, with a validation MSE of 0.0029289.
18	No significant improvement was observed compared with 15 neurons.
20	The model complexity increased, while the validation performance did not improve further.

**Table 4:** Quantitative comparison between BP and SSA-BP models on the independent test set.

Output Variable	Model	R	RMSE/mm	MAE/mm	MAPE/%	RMSE Reduction/%	MAE Reduction/%	MAPE Reduction/%
Penetration depth	BP	0.9441	0.0512	0.0391	3.85	-	-	-
Penetration depth	SSA-BP	0.9430	0.0439	0.0268	2.89	14.3	31.5	24.9
Weld width	BP	0.9492	0.0814	0.0624	4.94	-	-	-
Weld width	SSA-BP	0.9526	0.0561	0.0377	3.28	31.1	39.6	33.6
Overall	BP	0.9611	0.0680	0.0508	4.39	-	-	-
Overall	SSA-BP	0.9643	0.0504	0.0323	3.09	25.9	36.4	29.6

**Table 5:** Five-fold cross-validation results of the SSA-BP model using the original experimental samples.

Fold	Overall R	RMSE-Depth/mm	RMSE-Width/mm	MAE-Depth/mm	MAE-Width/mm	Overall MAPE/%
1	0.9920	0.0252	0.0200	0.0195	0.0161	1.66
2	0.9763	0.0289	0.0428	0.0229	0.0324	2.30
3	0.9853	0.0492	0.0327	0.0338	0.0255	2.82
4	0.9937	0.0228	0.0219	0.0191	0.0174	1.75
5	0.9912	0.0265	0.0331	0.0203	0.0226	1.88
Mean ± SD	0.9877 ± 0.0071	0.0305 ± 0.0107	0.0301 ± 0.0093	0.0231 ± 0.0062	0.0228 ± 0.0066	2.08 ± 0.48

**Table 6:** Parameter of experiment and result.

No.	Continuous Laser Power (kW)	Speed mm/min	Pulse Frequency (Hz)	Pulse Width (ms)	Width (mm)	Penetration (mm)	Ratio (Φ)
1	0.8	2000	40	5.5	0.97	0.8	0.82
2	0.9	2000	40	5.5	1.09	0.91	0.83
3	1	2000	40	5.5	1.21	1.02	0.84
4	1.1	2000	40	5.5	1.31	1.11	0.85
5	1.2	2000	40	5.5	1.43	1.15	0.8
6	1.3	2000	40	5.5	1.55	1.19	0.77
7	1.4	2000	40	5.5	1.66	1.22	0.73
8	1	1400	40	5.5	1.39	1.18	0.85
9	1	1700	40	5.5	1.27	1.09	0.86
10	1	2000	40	5.5	1.19	1.01	0.85
11	1	2300	40	5.5	1.12	0.92	0.82
12	1	2600	40	5.5	1.07	0.86	0.8
13	1	2900	40	5.5	1.04	0.82	0.79
14	1	3200	40	5.5	0.98	0.78	0.8
15	1	2000	40	4	1.11	0.93	0.84
16	1	2000	40	4.3	1.13	0.95	0.84
17	1	2000	40	4.6	1.16	0.96	0.83
18	1	2000	40	4.9	1.18	0.98	0.83
19	1	2000	40	5.2	1.19	0.99	0.83
20	1	2000	40	5.5	1.2	1.01	0.84
21	1	2000	40	5.8	1.21	1.03	0.85
22	1	2000	40	6.1	1.24	1.05	0.85
23	1	2000	40	6.4	1.26	1.06	0.84
24	1	2000	20	5.5	1.06	0.89	0.84
25	1	2000	25	5.5	1.1	0.92	0.84
26	1	2000	30	5.5	1.14	0.95	0.83
27	1	2000	35	5.5	1.18	0.98	0.83
28	1	2000	40	5.5	1.2	1.01	0.84
29	1	2000	45	5.5	1.22	1.04	0.85
30	1	2000	50	5.5	1.25	1.07	0.86
31	1	2000	55	5.5	1.28	1.09	0.85
32	1.1	2000	45	5.8	1.35	1.14	0.84
33	1.16	1400	50	6.1	1.63	1.24	0.76
34	1.2	1100	55	6.1	1.74	1.27	0.73
35	1	2000	40	5.5	1.21	1.02	0.84
36	0.9	2600	40	5.5	0.95	0.77	0.81
37	0.85	2900	35	5.2	0.77	0.68	0.88
38	1	2900	40	6.1	1.04	0.85	0.82
39	1	1100	30	5.8	1.41	1.19	0.84
40	1.05	1700	47	5.7	1.87	1.18	0.63

**Table 7:** Experimentally validated parameter sets and corresponding UTS values.

No.	Continuous Laser Power (kW)	Speed (mm/min)	Pulse Frequency (Hz)	Pulse Width (ms)	Ratio (Φ)	Ultimate Tensile Strength (MPa)
1	1	2300	40	5.5	0.82	1211.4
2	1	2000	30	5.5	0.83	1215.7
3	1	1100	30	5.8	0.84	1264.8
4	1.05	1700	47	5.7	0.63	796.0
5	0.85	2900	35	5.2	0.88	1061.1
Base metal	Not applicable	Not applicable	Not applicable	Not applicable	Not applicable	1400.1

Note: The base metal was not welded; therefore, welding parameters and weld aspect ratio are not applicable.

## Data Availability

The data presented in this study are available on request from the corresponding author.

## Abbreviations

The following abbreviations are used in this manuscript:

SSA	Sparrow Search Algorithm
BP	Back Propagation
SSA-BP	Sparrow Search Algorithm-optimized Back Propagation
EBSD	Electron Backscatter Diffraction
CW	Continuous Wave
MSE	Mean Squared Error
UTS	Ultimate Tensile Strength
HAZ	Heat-Affected Zone
